# The biomechanical evaluation of magnetic forces to drive osteogenesis in newborn’s with cleft lip and palate

**DOI:** 10.1007/s10856-020-06421-6

**Published:** 2020-08-20

**Authors:** Prasad Nalabothu, Carlalberta Verna, Markus Steineck, Andreas Albert Mueller, Michel Dalstra

**Affiliations:** 1 Department of Paediatric Oral Health and Orthodontics, University Center for Dental Medicine UZB, Basel, Switzerland; 2 Department of Oral and Craniomaxillofacial Surgery, University Hospital Basel, Basel, Switzerland

## Abstract

This study examined the potential for dental magnets to act as a driving force for osteogenesis in the palate of newborns with a unilateral cleft lip and palate. In the first part of the study dental magnets were arranged in a set up mimicking a distraction device and the curves of the magnetic attraction force versus gap distance curves generated, with and without the presence of palatal rugae tissue in between both sides of the distraction device. The attraction forces ranged from 1 to 12 N depending on the gap distance and the presence of soft tissue in the gap. In the second part of the study these forces were used as input for a 3D finite element model of the palate of a newborn affected by unilateral cleft lip and palate. In the analysis of load transfer, it was found that the strains generated by a magnetically induced distraction exceed 1,500 µstrain suggesting that bone locally is submitted to mild overload leading to bone apposition.

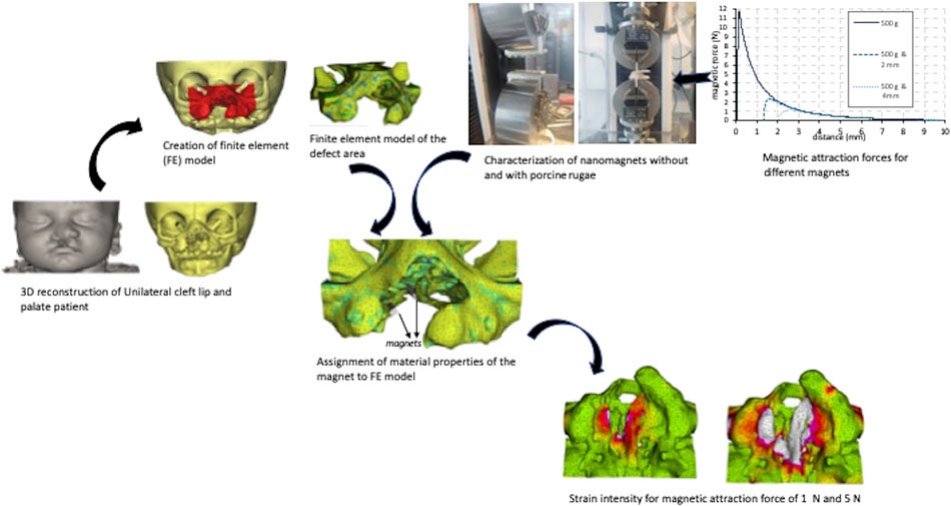

## Introduction

Cleft lip and palate (CL/P) is the most common presenting congenital condition of the face and cranial bones[1]. It is the most frequent craniofacial malformation affecting 1:500 to 1:700 live births worldwide with tremendous variations across geographic areas, ethnic groups and socioeconomic status [2]. Cleft palate closure by surgery depends upon the adequacy of available palatal mucosal tissue. Insufficiency of such soft tissue is a cause for concern in the closure of severe wide clefts of the hard palate [3]. The tissue shortage makes it necessary to extensively mobilize the tissue adjacent to the cleft, leading to a wider tissue wound with consequent scarification due to the wound healing process. During wound repair, various tissues are involved, including palatal skeletal muscle, skin and mucosa. Inadvertently, the CL/P surgery leaves scars, which may interfere with normal growth and development of the midface and dentition [4].The other complications include insufficient palatal muscle function, which hampers swallowing, sucking and speech [5].

Tissue expansion techniques, which have gained popularity in other areas of the body for reconstructive needs, have been proposed for cleft palate repair [6]. The tissue expansion techniques described for cleft palate repair are categorized into two broad categories: mucoperiosteal tissue expansion (MTE) and distraction osteogenesis (DO). The procedure of MTE intends to expand purely the soft tissue overlying the bone. It was initially employed for fistula closure and later extended for the repair of primary cleft palate [7]. The principle employed is that of an expander placed under the mucosa on either ends of the palatal defect in an effort to generate additional soft tissue that can be used to close the defect. However, on a contrary note seven out of eleven patients had fistulas and some of the patients were found to require premature removal of the tissue expanders due to cyanosis or pale tissue [7]. So far, the results seem to raise many doubts and dilemmas and its effectiveness is still questionable.

Distraction osteogenesis (DO) is a method of increasing bone volume by controlled daily separation of the bone ends on either side of a surgical bone cut. The increase in bone volume leads to expansion of its overlying soft tissue. Callus formation occurs after 5 to 7 days, followed by bony ingrowth. When the required bone length has been achieved, the distraction device remains in place to serve as a rigid skeletal fixation until maturation of the generated new bone is achieved in the consolidation period [8]. Till date, there is to our knowledge only one case report of distraction treating palatal fistula in adults [9] yet unfortunately it provides little evidence supporting the efficacy of DO in cleft palate repair.

The periosteal distraction osteogenesis (PDO) is a variation of DO, where the gradual tissue separation is made between the periosteum and the bony surface, allowing at the same time for new bone formation without corticotomy [10] and expansion of the overlying soft tissues, such as periosteum, muscle and skin.

Static magnetic fields have been used for bone formation in medical research under a weak magnetic force for a long period [11]. To understand the mechanism of the mucoperiosteal tissue expansion and tissue formation, the distribution of stresses and strains within the periosteal tissue is required.

Computational Finite Element (FE) modelling of the tissue provides a quantified estimate of the stresses and strains generated by a magnetic force through the mucoperiosteal tissue to the underlying bone. The irregular geometry of the periosteal tissue and microarchitecture of the underlying bone in the unilateral cleft models has been simplified in previous models, despite the fact that such differential anatomical features can significantly influence the spatial stress distribution in the palate [12]. To date there is no study investigating the mechanical loading during periosteal tissue expansion in cleft palate of newborns with unilateral clefts.

The objective of this study is to quantify the stresses and strains in the palate of a newborn with a unilateral cleft lip and palate (UCLP) simulating the periosteal loading through magnetic forces and to verify whether the loading could reach the threshold necessary for bone formation. The early formation of new bone would reduce the gap and therefore the strain induced by the surgical closure. As at this stage no *in-vivo* load transducers can be placed, patient-specific finite element modelling is the obvious approach to assess the magnetic load transfer across the neonatal palate.

## Materials and methods

The study consisted of two parts. Firstly, the magnitude of dental magnets in various implant-like *in-vitro* setups were measured. Data from these experiments were subsequently used as input for a 3D FE-model of the cleft palate of newborn to assess the palatal load transfer.

### Experimental characterization of the dental magnets

**Fig. 1:**
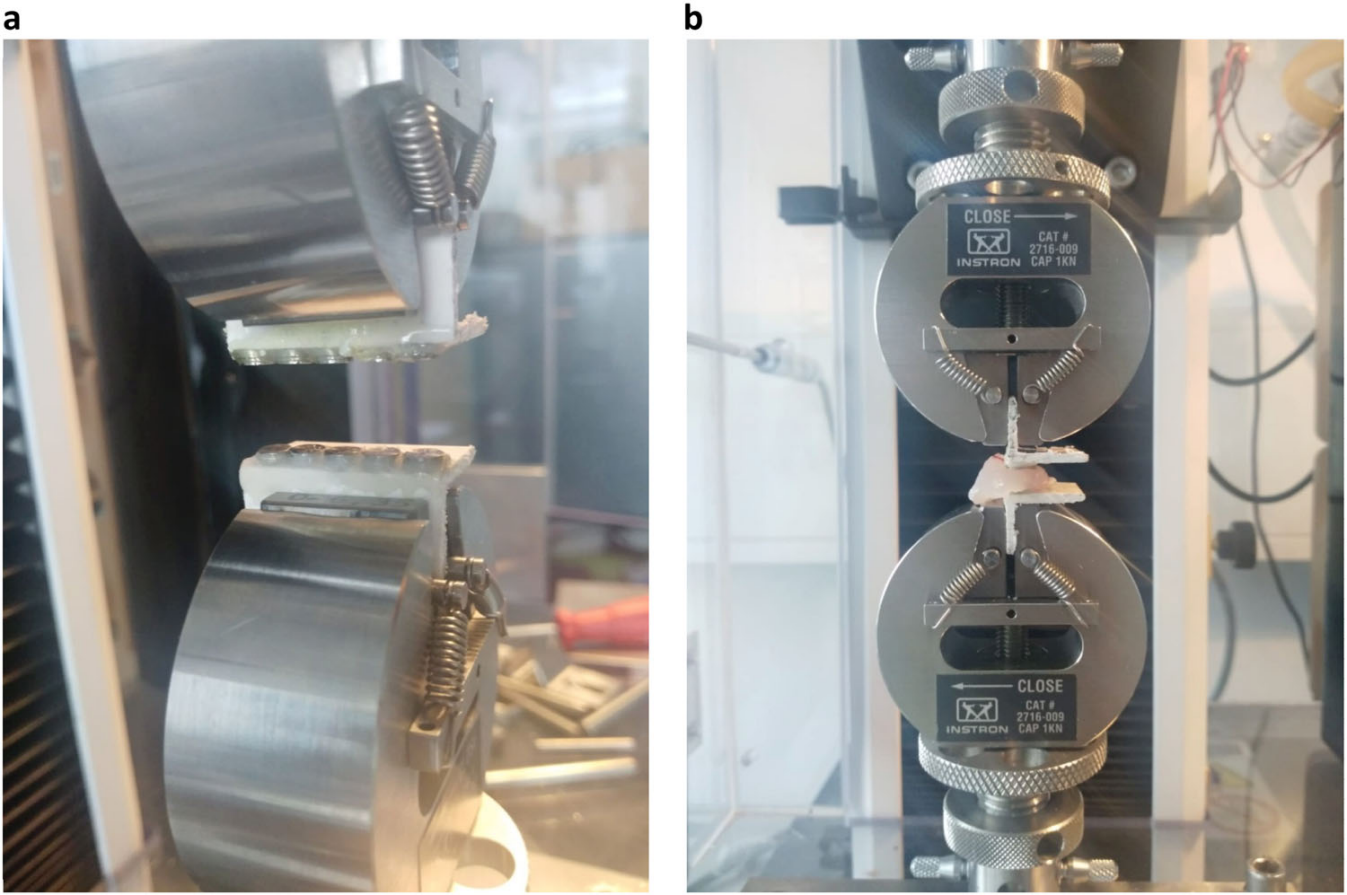
**a** Magnets mounted in strips for testing on Universal material testing machine (Instron, Norwood, Massachusetts, USA). **b** Porcine rugae tissue with a thickness of 4 mm between the magnets

Two different types of magnets were used for testing (Dyna WR magnets, Dyna Dental Engineering B.V., Halsteren, Netherlands). The dental magnets were disc-shaped with dimensions: 1.7 mm (02MS1 Dyna WR magnet S3) and 2.7 mm height (02MS2 Dyna WR magnet S5), both with 4.5 mm diameter. The 1.7 mm magnet (S3) had a nominal attachment force of 300 g, whereas the 2.7 mm magnet (S5) had 500 g. The magnets are made of Neodymium/Boron/Iron alloy and belong to the group of rare earth or permanent magnets. The magnets were glued onto two plastic strips in five configurations (a single magnet, two in a row, etc. up to five in a row). Each strip was mounted into the opposing grips of a universal material testing machine (Instron^®^ 3344 equipped with a 100 N load cell; Instron, Norwood, Massachusetts, USA) (Fig. 1a). The initial distance between the opposing strips with magnets was 10 mm and this gap was closed by the testing machine at a speed of 10 mm/min. A continuous readout of the attraction force values and gap distance was recorded, enabling the construction of force/distance plots. The experiments were repeated with a 2 mm and 4 mm thick slice of porcine palatal rugae tissue (obtained from a local butcher shop), placed between the magnets (Fig. 1b), simulating the fact that the magnets, once in use, would be implanted subcutaneously.

### Creation of the FE-model

**Fig. 2:**
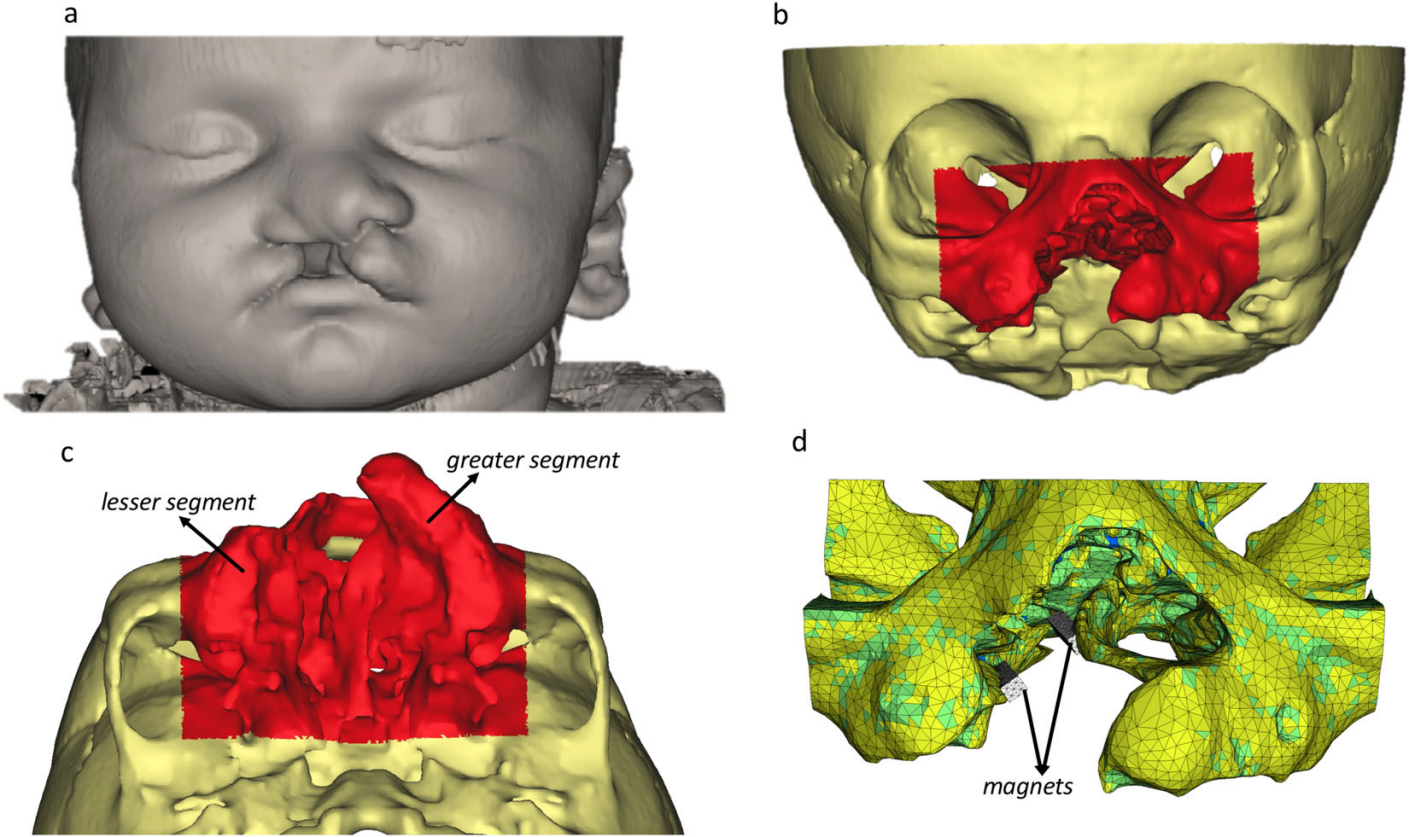
From CT scan to FE-model. **a** The 3D reconstructions of the soft and hard tissues, **b** Frontal view of the region of interest for the FE model **c** Palatal view of the region of interest for the FE model **d** and the FE model of the dental magnet partially visible

A computerized tomography (CT) scan of a 2-week old newborn with a unilateral cleft lip and palate from an anonymized database following the principles expressed in the Declaration of Helsinki was made available. The scans were imported into dedicated image analysis software (Mimics v.19, Materialise, Leuven, Belgium) and a 3D surface model was generated by thresholding and selecting only the hard tissues of the cranium and maxilla (Fig. 2a). The region of interest was further reduced by virtually cutting away everything except the mid-face (Fig. 2b, c). Two sides of a bone distraction/generation implant were constructed as two parallel bars (5 × 6 × 23 mm^3^) and each was placed at either side of the cleft (Fig. 2d). Using the program’s FE module, the model, including the two implant bars, was transformed into a 3D FE-mesh consisting of 238,243 4-node tetrahedral elements with 43,822 nodes. The position of the centroid of each element was calculated and a grey value was associated to it, based on spatial interpolation of the grey values of the nearest voxels. Using arithmetic relationships between grey values, apparent densities and Young’s moduli, relevant material properties were assigned to the elements [12–15] (Table 1). Subsequently, the Young’s moduli were stratified into ten material property groups, ranging from 5.2 MPa, representing very low-density cancellous bone (ρ_app_ = 0.04 gr/cm^3^) to 18,050 MPa, representing high density cortical bone (ρ_app_ = 1.90 gr/cm^3^). The FE-model was then exported from the image analysis program in NASTRAN format and imported into an FE-analysis program (Strand7, Sydney, Australia). The material properties of the bone were assumed non-linear, as the stress-strain curves for the bone in the ten material property groups were adapted such that the bone would behave as a perfectly plastic material with a 150 per cent higher compressive strength than a tensile strength[16].

**Table 1:** Mechanical properties of the materials used in the present study. For the different bone tissues (1 until 10) the percentage signifies the cumulative amount of bone elements belonging to this specific tissue

Material	Apparent Density (gr/cm^3^)	Young’s Modulus (MPa)	Poisson’s Ratio (-)	Yield Strength	
				Compressive (MPa)	Tensile (MPa)
Bone 1 (0.1%)	0.04	5	0.4	−0.06	0.04
Bone 2 (0.5%)	0.10	51	0.4	−0.61	0.40
Bone 3 (30.9%)	0.18	157	0.4	−1.89	1.26
Bone 4 (89.6%)	0.26	351	0.4	−4.22	2.81
Bone 5 (97.8%)	0.36	677	0.4	−8.12	5.41
Bone 6 (98.8%)	0.50	1212	0.4	−14.54	9.70
Bone 7 (99.4%)	0.66	2115	0.4	−25.37	16.92
Bone 8 (99.8%)	0.83	3752	0.4	−45.02	30.02
Bone 9 (99.9%)	1.20	7226	0.4	−86.72	57.81
Bone 10 (100%)	1.90	18048	0.4	−216.58	144.38
Implant	–	100	0.4	–	–

### Preprocessing and FE-analysis

Prior to the analysis the boundary conditions were defined by restraining the degrees of freedom of the nodes on the cut border surfaces perpendicular to these surfaces of the model. The nodes were allowed to move in the plane of these border surfaces to simulate residual stiffness of the (non-modeled) rest of the skull.

External forces were assigned to the two facing sides of either bar implant. The force magnitude derived from the abovementioned experiments with the dental magnets, while the direction followed from the direction of mutual attraction.

The FE-analysis was conducted with the abovementioned material considerations, boundary conditions and external loading, using a non-linear solver with logarithmically increasing increments from 0.01 to 1 (100% load). The various components and invariants of the deformations, stresses and strains were calculated and used for subsequent post-processing.

## Results

### Experimental characterization of dental magnets

**Fig. 3:**
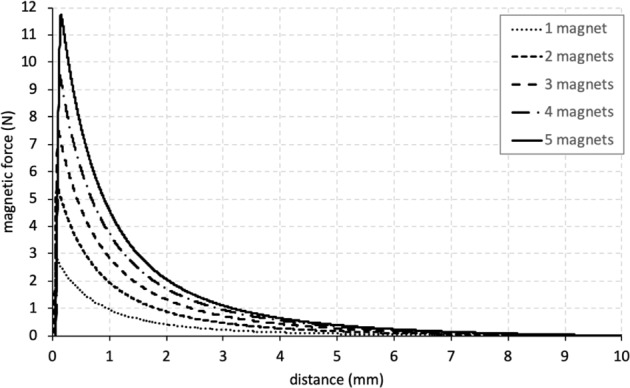
The magnetic attraction forces as a function for the distance between the magnets for one until five magnets

**Fig. 4:**
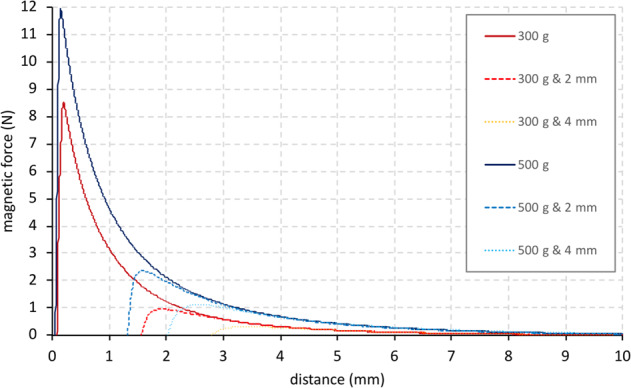
The magnetic attraction forces for five 300 and 500 gram magnets with either no or 2 mm thick or 4 mm thick porcine rugae

The experimental force/distance curves of each magnet with different combinations were obtained. The combination of two oppositely placed single 500 g magnets yielded a maximum attraction force of 2.8 N, right before the two magnets came in contact with each other and compressive forces started to develop. Adding up to 5 magnets on either side of the testing device increased the attraction force to 11.7 N (Fig. 3). The distance between the magnets, however, had a significant influence on the magnitude of the attraction force. When the separation between the magnets is 1 mm the attraction forces are merely 1.0 and 4.6 N for a set of one and five opposing magnets, respectively. Adding a layer of porcine palatal rugae tissue in itself did not influence the test curves, as long as the two sides were sufficiently apart. However, the presence of rugae tissue prevented the magnets to come closer together, which in turn prevented the attraction forces to reach levels seen for the curves without rugae tissue present (Fig. 4).

### Numerical assessment of palatal loading through dental magnets

**Fig. 5:**
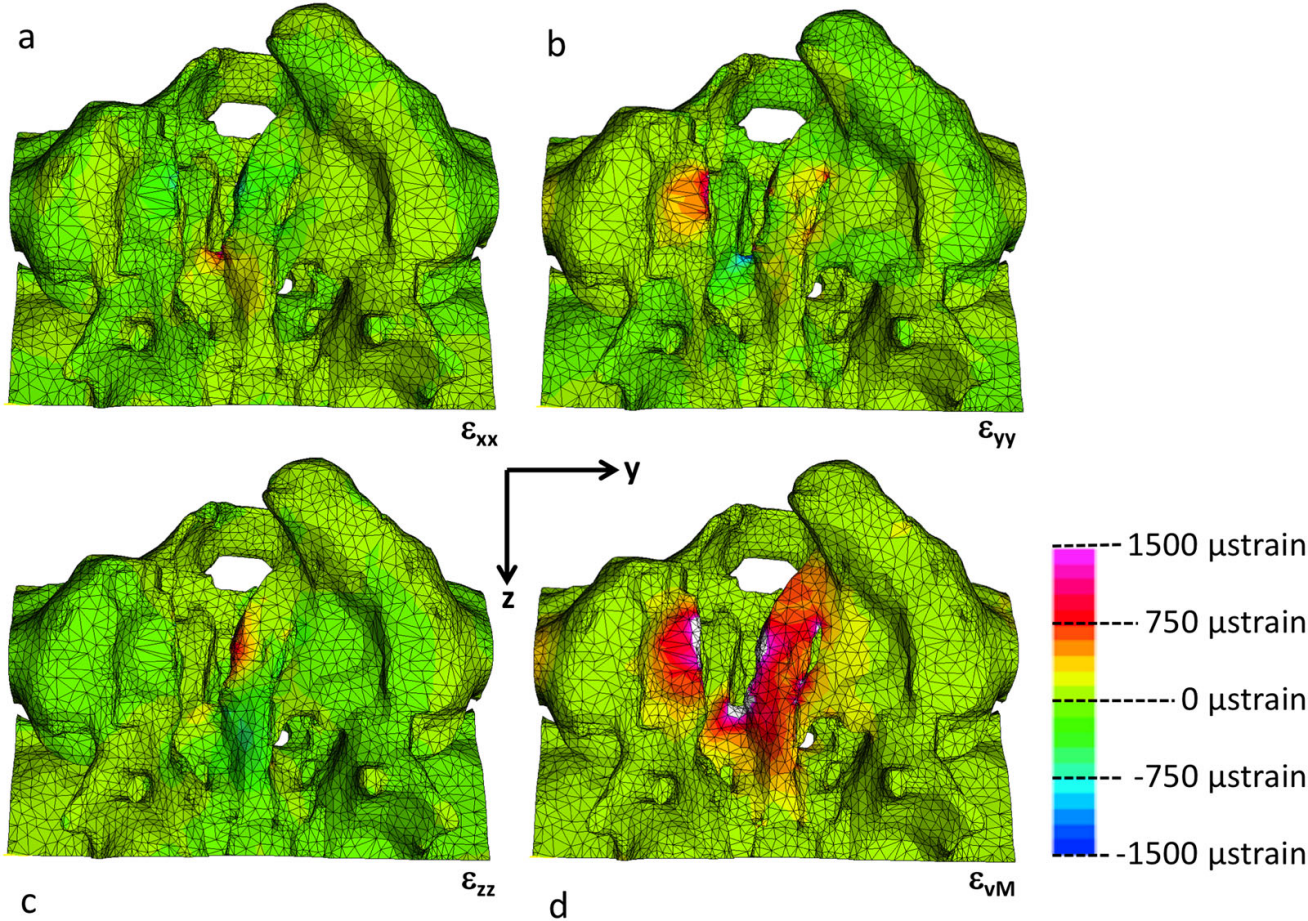
The distribution of the three orthogonal strain components von Mises strains for magnetic attraction forces of 1 N **a** palatal view with the X-axis pointing coronally, **b** Y-axis pointing left, **c** Z-axis pointing posteriorly and **d** the strain intensity (von Mises strain)

The strain components in the X- (coronal), Y-(medial/lateral) and Z- (anterior/posterior) are different in each direction. The application of magnetic attraction forces of 1 N to the implant bars of the FE-model would represent the force level corresponding to the presence of rugae tissue at a separation of 3 to 4 mm between the two sides of the implant design, assuming the presence of five 500g-magnets. In the Y-direction, which approximates closely the direction of the magnetic forces, the strains are predominantly localized in the palatal shelf of the lesser segment and vomer edge (Fig. 5b). In the X- direction, the largest strains are concentrated in the posterior part of the vomerine edge of the cleft (Fig. 5a), while in the Z- direction the strains are mainly seen in the middle portion of greater segment’s palatal shelf ridge (Fig. 5c). The distribution of the von Mises strains, a measure for the overall strain intensity, showed the largest values on both sides of the cleft and reached values between 750 and 1,500 µstrain (Fig. 5d).

**Fig. 6:**
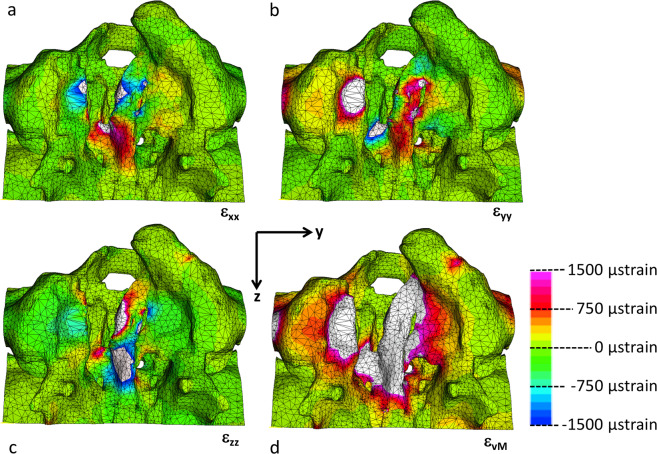
The distribution of the three orthogonal strain components von Mises strains for magnetic attraction forces of 5 N **a** palatal view with the X-axis pointing coronally, **b** Y-axis pointing left, **c** Z-axis pointing posteriorly and **d** the strain intensity (von Mises strain)

The application of an attraction force of 5 N exceeded the experimental results with rugae tissue present and would thus represent an overestimation of the magnetic forces. In this case, the strain levels exceeded 1,500 µstrain. For the coronal (X-) direction the strains exceeding 1,500 µstrain are found in the vomerine ridge, in the palatal shelf of the lesser and in the greater segments of palatal shelf ridge (Fig. 6a). In the medial/lateral (Y-) direction the maximum strains are found on the palatal shelf of the lesser and greater segment and on the vomerine surface (Fig. 6b) and in the antero-posterior (Z-) aspect, the maximum strains are found on the greater segment of the palatal shelf ridge (Fig. 6c). The highest von Mises strains are found on both sides of the palatal cleft segment (Fig. 6d).

**Fig. 7:**
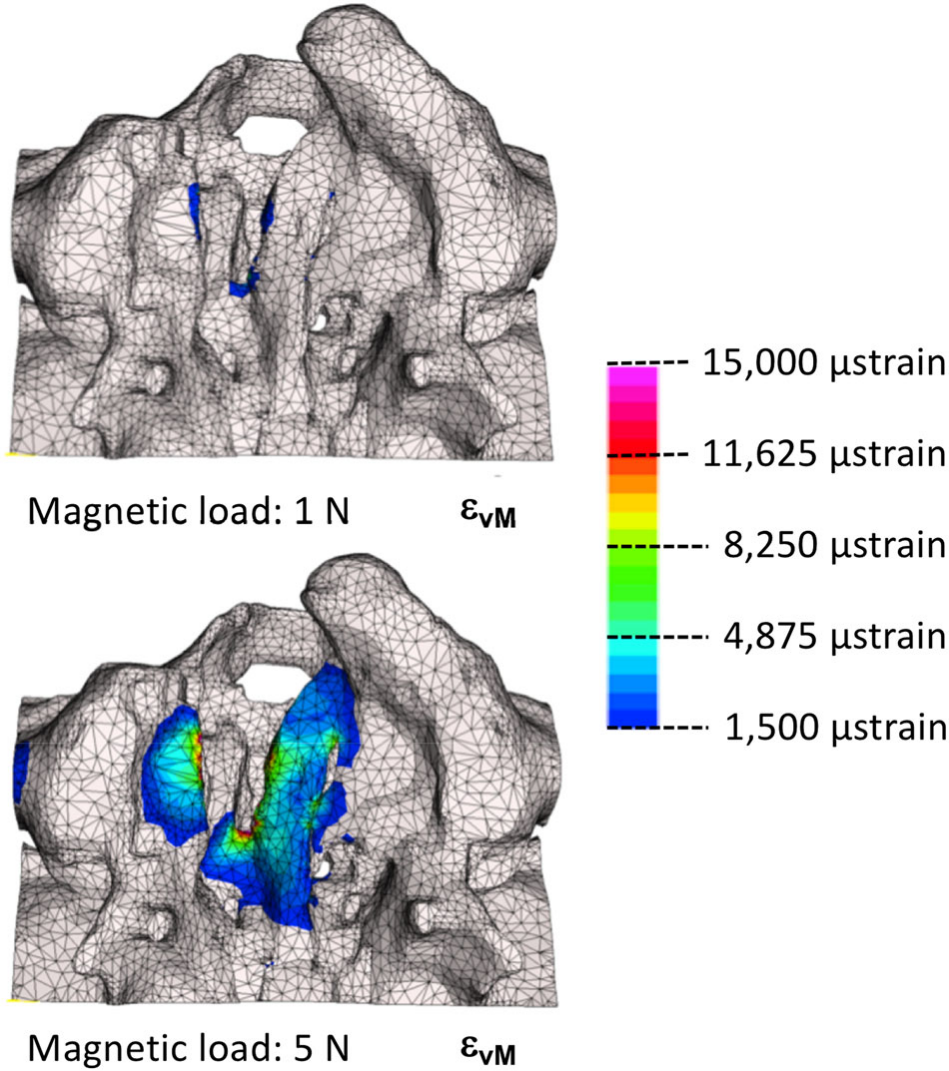
The distribution of the von Mises strains for magnetic load of 1 N and 5 N in the range of 1,500 to 15,000 μstrain (palatal view with the X-axis pointing coronally, Y-axis pointing left and Z-axis pointing posteriorly). Note that the non-colored regions are not subject to adaptive remodeling

Visualizing in more detail at where in the model the strain levels are >1,500 µstrain, it can be seen that these occur predominantly in the direct vicinity of the cleft (Fig. 7). Although for the magnetic force of 1 N this limit has just been reached, the 5 N magnetic force caused a substantially larger area of periosteal tissue to be strained at >1,500 µstrain.

## Discussion

The aim of this study was to assess whether dental magnets have the potential to act as a device to generate palatal bone tissue. Harold Frost hypothesized, in his Mechanostat theory, that the amount of tissue being formed (or resorbed) is dependent on the actual load level it is subjected to: a) Bone strained with less than 1,000 µstrain is in a state of disuse, which could lead to bone resorption and net bone loss [17–19]. b) Bone with 1,000–1,500 µstrain is in an adaptive state, whereby no bone is either lost or gained. c) Bone strained to more than 1,500 µstrain is in a state of mild overload and new bone will be formed. d) Finally, towards the upper limit of this window (15,000 µstrain) a case of severe overload might occur with the risk of fracture. Therefore, if a minimum strain level of 1,500 µstrain is to be achieved for bone apposition, a detailed insight in the palatal load transfer mechanism in newborns with cleft lip and palate is required.

To see whether dental magnets can generate sufficiently high forces, several set-ups were tested, including the absence or presence of rugae tissue between the magnets. For this, palatal rugae samples were excised from a pig’s head, obtained at a local butchery, as porcine soft tissue is generally seen as to come close to the human equivalent. Depending on the number of magnets and the inter-magnet distance, attraction forces in the order of magnitude of 1 to 10 N were measured. It should be noted here, however, that the experimental set-up is not meant as the design for a prototype of the palatal distraction device but only to establish the force/distance relationship.

Due to ethical reasons, strain-measuring devices or force transducers cannot be implanted in newborns with a cleft to assess the palatal load transfer at this stage. Therefore, an in*-silica* approach in the form of a detailed 3D FE-model was considered the only valid alternative for which a scan of an actual patient with unilateral cleft lip and palate was required. The scan, which was made available, had originally been taken for a medical condition (hydrocephaly) and it was justified and proven that this condition did not cause deformity in craniofacial growth and morphology [20], Provided an FE-model is well designed and constructed and its assumptions are well-founded, it can supply results which cannot be obtained through other means. Within orthopedics FE-modelling is a well-established and generally accepted method to analyze implant-to-bone load transfer and to support the design of new implants, often using patient-specific models [21]. In our study, 3D data from CT scans were used to create a geometrically accurate model of the palatal region of a newborn with a unilateral cleft lip/palate. Furthermore, the spatial distribution of the grey values in the 3D data-set was used to provide the elements with material properties approximating the actual distribution of bone stiffness in the facial skeleton. Direct measurements of Young’s modulus and other material properties of neonatal palatal bone are difficult to perform for both technical and ethical reasons. However, studies are available in which the relationships between grey values of CT data of bone, and its density and Young’s modulus. In the present study, it was assumed that the highest grey values corresponded to the presence of a high-density cortical bone with a Young’s modulus of 18 GPa. For the association of other grey values and bone stiffness, the relationships given by [13–15] were scaled to fit the 18 GPa upper limit.

As no information of the force levels for the facial musculature of newborns was available and tongue thrust and sucking forces are irregular, both in magnitude and frequency, it was decided only to incorporate the magnetic attraction forces of the virtual palatal implant in this study. It should thus be kept in mind that in reality the stresses and strains due to the magnetic distraction device would have to be added to those generated by the aforementioned physiological sources.

In the present study we found that magnetic forces of dental magnets range from 1–10 N in an implant- like set up. The strain levels in the palatal segments of the cleft for these load cases do reach the 1,500 µstrain limit for mild overuse, suggesting that periosteal tissue modelling will be induced. The amount of separation between the two sides of the implant will be important for the magnitude of the magnetic forces. Especially for a narrow gap between both sides of the implant generate large forces, yet a mere millimeter larger gap would see substantially lower attraction forces. As for a working distraction device both sides (greater and lesser segments of the cleft palate) of the implant would gradually come closer to each other due to a newly generated bone, the attraction would become larger and higher strain levels would be incurred. The presence of soft tissue due to the subcutaneous design of the implant, however, will prevent the full contact of the opposing magnets and generate their maximum attraction force potential.

The directional strain components were largest in the medio/lateral aspect, thereby following the magnetic forces on either side of the implant. The anterior portion of the lesser segment featured the largest tensile strains, followed by the strains in the greater segment. On the posterior part of the vomer mediolateral strains were compressive in nature and are caused by squeezing action during the distraction process. However, these will depend on individual anatomical variations and size of the cleft. Due to Poisson effect the strains in the coronal aspect are opposite in nature to the aforementioned strains in the mediolateral aspect. To a lesser degree it also occurs in the anteroposterior direction.

As mentioned above, it was not the aim of the current study to present a prototype of a magnetic palatal distraction device for the use in newborns. It has merely confirmed the hypothesis that dental magnets do have the potential to generate levels of tissue strains, associated with mild overload, resulting in bone apposition. As the feasibility of dental magnets for the purpose of osteogenesis has now been demonstrated, the next step will be to produce a prototype design of the device, and test it in an ex-vivo and in-vivo animal model to validate the findings of the presented FE-analyses.

## Conclusions

1. Dental magnets can produce attraction forces large enough to be used for adaptive remodelling or guided bone formation on infant cranio-facial complexes.
2. The strains in the periosteum and the underlying palatal bone generated by magnetic attraction forces are compatible with adaptive bone formation in the unilateral cleft palate model.
3. With magnitudes exceeding 1,500 µstrains, the tensile strains in the lesser and greater segments will act as stimulus for guided bone formation in the medio-lateral aspect.

## References

[1] Mossey PA, Little J, Munger RG, Dixon MJ, Shaw WC (2009). Cleft lip and palate. Lancet.

[2] Mossey P (2003). Global strategies to reduce the healthcare burden of craniofacial anomalies. British Dental Journal.

[3] Latham RA, Smiley GR, Gregg JM (1973). The problem of tissue deficiency in cleft palate: An experiment in mobilising the palatine bones of cleft dogs. Br J Plast Surg.

[4] Shetye PR, Evans CA (2006). Midfacial morphology in adult unoperated complete unilateral cleft lip and palate patients. Angle Orthod.

[5] Monroy PLC, Grefte S, Kuijpers-Jagtman AM, Wagener FADTG, Von Den Hoff JW (2012). Strategies to improve regeneration of the soft palate muscles after cleft palate repair. Tissue Eng - Part B: Rev.

[6] Shash H, Al-Halabi B, Jozaghi Y, Aldekhayel S, Gilardino MS (2016). A review of tissue expansion-assisted techniques of cleft palate repair. J Craniofac Surg.

[7] Kobus KF (2007). Cleft palate repair with the use of osmotic expanders: a preliminary report. J Plast Reconstr Aesthetic Surg.

[8] McCarthy JG, Schreiber J, Karp N, Thorne CH, Grayson BH (1992). Lengthening the human mandible by gradual distraction. Plast Reconstr Surg.

[9] Alkan A, Baş B, Özer M, Bayram M (2007). Closure of a large palatal fistula with maxillary segmental distraction osteogenesis in a cleft palate patient. Cleft Palate-Craniofacial J.

[10] Schmidt BL, Kung L, Jones C, Casap N (2002). Induced osteogenesis by periosteal distraction. J Oral Maxillofac Surg.

[11] Yan QC, Tomita N, Ikada Y (1998). Effects of static magnetic field on bone formation of rat femurs. Med Eng Phys.

[12] Wang D, Cheng L, Wang C, Qian Y, Pan X (2009). Biomechanical analysis of rapid maxillary expansion in the UCLP patient. Med Eng Phys.

[13] McPherson GK, Kriewall TJ (1980). The elastic modulus of fetal cranial bone: A first step towards an understanding of the biomechanics of fetal head molding. J Biomech.

[14] Bauer FX, Heinrich V, Grill FD, Wölfle F, Hedderich DM, Rau A (2018). Establishment of a finite element model of a neonate’s skull to evaluate the stress pattern distribution resulting during nasoalveolar molding therapy of cleft lip and palate patients. J Cranio-Maxillofac Surg.

[15] Pan X, Qian Y, Yu J, Wang D (2007). Biomechanical Effects of Rapid Palatal Expansion on the Craniofacial Skeleton. Cleft Palate-Craniofacial J.

[16] Carter DR, Schwab GH, Spengler DM (1980). Tensile fracture of cancellous bone. Acta Orthop.

[17] Frost HM (2000). The Utah paradigm of skeletal physiology: An overview of its insights for bone, cartilage and collagenous tissue organs. J Bone Min Metab.

[18] Frost HM (2004). A 2003 update of bone physiology and Wolff s law for clinicians. Angle Orthod.

[19] Turner CH (1999). Toward a mathematical description of bone biology: The principle of cellular accommodation. Calcif Tissue Int.

[20] Yusof A Craniofacial growth changes in Malaysian Malay children and young adults: a cross-sectional 3-dimensional CT study. [Internet]. University of Adelaide; 2007. Available from: https://digital.library.adelaide.edu.au/dspace/handle/2440/39388.

[21] Poelert S, Valstar E, Weinans H, Zadpoor AA (2013). Patient-specific finite element modeling of bones. Proc Inst Mech Eng Part H J Eng Med.

